# What makes us human: revisiting an age-old question in the genomic era

**DOI:** 10.1186/1747-5333-1-18

**Published:** 2006-11-29

**Authors:** Nitzan Mekel-Bobrov, Bruce T Lahn

**Affiliations:** 1 Howard Hughes Medical Institute, Department of Human Genetics and Committee on Genetics, University of Chicago, Chicago, IL 60637, USA

## Abstract

In 1970, Karl Pribram took on the immense challenge of asking the question, what makes us human? Nearly four decades later, the most significant finding has been the undeniable realization of how incredibly subtle and fine-scaled the unique biological features of our species must be. The recent explosion in the availability of large-scale sequence data, however, and the consequent emergence of comparative genomics, are rapidly transforming the study of human evolution. The field of comparative genomics is allowing us to reach unparalleled resolution, reframing our questions in reference to DNA sequence – the very unit that evolution operates on. But like any reductionist approach, it comes at a price. Comparative genomics may provide the necessary resolution for identifying rare DNA sequence differences in a vast sea of conservation, but ultimately we will have to face the challenge of figuring out how DNA sequence divergence translates into phenotypic divergence. Our goal here is to provide a brief outline of the major findings made in the study of human brain evolution since the Pribram lecture, focusing specifically on the field of comparative genomics. We then discuss the broader implications of these findings and the future challenges that are in store.

## Background

In his seminal lecture in 1970, Karl Pribram asked the question, what makes us human? Nearly four decades of research has accumulated since, and major technical advances have been made, but our newly-gained perspective affords us little more than the realization of how exceedingly subtle and fine-scaled the unique biological features of our species must be. This is nowhere more evident than in studies of the human brain. Our evolutionary history has generated a vast repertoire of behavioral oddities, which to most stand out as entirely unique and remarkably divergent from other species, and to many is an obvious testament to our singular position in nature. Faced by our great lack of discernibly unique biological features, however, just how this evolutionary feat was achieved is anything but obvious.

Traditionally, studies of brain evolution have focused on comparative neuroanatomy. By the time of Darwin's *Origin of Species *in 1859, anatomical variation among species had been the subject of contentious debate for several decades [1]. Mirroring the philosophical rift of the early 19^th ^century, biologists of the time were divided between the rationalists' functional taxonomy and a structural morphology based on generalized archetypes. Darwinian evolution provided a theoretical framework that accounted for both form and function, by bringing together heredity and adaptation under the single umbrella of natural selection [2]. Championed by the studies of Thomas Huxley, Darwinian theory quickly came to dominate interpretations of comparative neural anatomy, and the field of evolutionary neurobiology was born. Advances in microscopy around the turn of the century propelled the field forward, providing key insights into interspecies variation in the structural compartmentalization of the brain. As progress in histological techniques continued through most of the twentieth century, studies in comparative neural anatomy generated invaluable data on variation in both cellular organization and subcellular neuronal components. Thus, the study of brain evolution, from its inception, has been entrenched in the tradition of comparative anatomy.

Comparative anatomy on its own, however, has relatively limited resolution, unable to distinguish between many neuronal subtypes or identify small-scale structural variation. In 1970, when Pribram was calling for a biological definition of the uniquely human cognitive process of transforming experience into meaning, the life sciences were on the verge of a revolution. With major technological advances ushering in the molecular era of biology, the study of brain evolution has been integrating new high-resolution approaches. New data on gene expression, allowing finer classification of neuronal subtypes and histogenic fields, have revealed detailed homologies among various subdivisions of the vertebrate brain [3-5]. The advent of targeted mutagenesis, gene knockdown, and transgenic technologies in a wide range of model organisms has begun to elucidate the complex interplay between evolutionary conservation and the emergence of novel functions [6]. Perhaps most importantly, after a century of fission between evolutionary biology and embryology, advances in developmental genetics have sparked a renewed interest in the role of ontogeny in phylogenetic change. More nuanced than Haeckel's biogenic law of ontogeny recapitulating phylogeny, the new evolutionary developmental biology views development as the substrate of evolutionary change [7]. Thus, the study of brain evolution today is markedly different from its predecessor four decades ago. This transformation marks a true paradigm shift; the very concepts of conservation and divergence now redefined in the context of molecular phenotypes and developmental processes.

Whereas the overall field of evolutionary neurobiology has undergone a major transformation over the past few decades, the study of human brain evolution has remained largely unchanged. This discrepancy is due to the technical and ethical limitations inherent to any study of human subjects. This observation is underscored by the tremendous focus still placed in studies of human brain evolution on broad issues of scaling and allometry [8]. A major challenge, therefore, is to find alternative approaches for enhancing our resolution of interspecies variation.

One such approach is the field of comparative genomics. The last decade has seen an explosion in the availability of large-scale sequence data for both human and nonhuman species. The first publication of the human genome in 2001 heralded the long-awaited arrival of the genomic era, and with many other genome projects either completed or underway, the newly emergent field of comparative genomics has rapidly become a major staple of evolutionary analysis. This new approach affords us unparalleled resolution, as the study of molecular divergence, by definition, gets at the most fundamental level of evolutionary change. Though still in its infancy, the field of comparative genomics has already made important progress in probing the genetic basis of what makes us human. Our goals here are to provide a brief outline of these findings; to discuss their broader implications for the study of human brain evolution; and ultimately, we hope, to demonstrate the utility of this approach in narrowing the gap between human and nonhuman evolutionary neurobiology.

### Comparative genomics and the unique features of the human genome

The first publication of the human genome in 2001 [9] marked a turning point in the study of human biology. It was only with the availability of large-scale sequence data from other vertebrate species, however, that we could begin a comparative approach, to identify those features of our genome, which potentially underlie the unique human phenotype. Studies in comparative genomics, on several taxonomic levels, have made great strides in identifying the important features of our lineage, shared by virtue of being vertebrates, mammals, primates, anthropoids, etc' [10-13]. But the recent genome publication of our closest living relative, the chimpanzee [14], coupled with large-scale data from various outgroup species, now allows us for the first time to identify the features of our genome that are truly unique to our species, and are, as such, the building blocks of the distinct human phenotype.

Comparisons of the human and chimpanzee genomes have identified 35 million single nucleotide substitutions, roughly a 1.23% single nucleotide divergence [14]. After removing the portion of substitutions showing intraspecies polymorphism, the human-chimp single nucleotide divergence is estimated at roughly 1%. As had been previously observed at specific loci [15], however, insertion and deletions (indels) are major contributors to the human-chimpanzee divergence. Roughly 90 Mb in total, indels raise the overall human-chimpanzee divergence to approximately 4%, significantly higher than most previous estimates [16,17]. Curiously, the human insertions do not appear to be randomly distributed across the genome, but rather clustered together on a subset of chromosomes. Whether this positional bias has some functional significance, or is strictly the result of their evolutionary history, remains to be seen. Nonetheless, in regards to protein-coding sequences, the human-chimpanzee divergence is less than 1%, with the average protein differing by only two amino acids.

The human and chimpanzee genomes show structural differences as well, ranging in scale from local events to large-scale chromosomal alterations. Sequence inversions between humans and chimpanzees are estimated at over 1500 events, ranging in size from 23 bp to as large as 62 Mb [18]. Cheng *et al. *identified 296 regions in the human genome showing significant copy number increases in humans compared to chimpanzees. These segmental duplications span 7.2 Mb and are preferentially located in pericentromeric regions and on chromosomes 5 and 15 [19]. Identifying segmental duplications, though long thought to be a key mechanism for generating evolutionary novelty [20], poses a serious technical challenge, since the method used to generate the chimpanzee draft sequence has considerable difficulty in distinguishing between highly similar sequences [21]. To address this problem, Locke *et al. *developed an array-based comparative genomic hybridization method; using an array of 2,460 human bacterial artificial chromosomes (BACs), they demonstrated the utility of this approach in identifying deletions and duplications that cannot be resolved by whole-genome shotgun sequencing [22]. Wilson *et al. *subsequently adopted this approach, using a whole-genome human BAC array. They identified 63 chromosomal segments showing increased copy number in humans relative to chimpanzees, ranging in size from 0.65 to 1.3 Mb, and spanning almost 200 genes [23]. Alternatively, cDNA arrays have also been used. Using this approach, Fortna *et al. *identified 1,005 genes showing variation in copy number among hominoid lineages, and found that copy number expansions were most pronounced in humans, with 134 genes showing increased copy number and only six showing copy number decrease [24]. Finally, repetitive elements such as LINEs (long interspersed elements) and SINEs (short interspersed elements) can spread and expand throughout the genome by reverse transcription, resulting in potentially important modifications to either coding or regulatory sequences [25]. Comparison of repetitive elements between humans and chimpanzees has shown that *Alu *elements, the most common type of SINEs in humans [26], have expanded in the human genome to a frequency three times higher than that of chimpanzees [14].

Genomic comparisons with our closest living relative is a necessary first step towards identifying human-specific genomic features, but the addition of informative outgroup species is essential for narrowing down these differences to those that are uniquely human, rather than uniquely chimpanzee. The choice of outgroup species, however, is not always straightforward, as it can have considerable impact on one's findings. In a comparison of 7,645 orthologous genes between humans and chimpanzees, Clark *et al. *used the mouse as the outgroup species [27]. The long divergence time between rodents and primates introduced considerable error in inferring the ancestral states between the human and chimpanzee sequences, particularly at rapidly evolving sites. Consequently, in their choice of an outgroup, Yu *et al. *used a primate species instead, finding that in human-chimpanzee comparisons, the macaque monkey performed much better than the mouse [28]. Thus, as additional primate genome sequences become available, our power to detect human-specific genomic features will demonstrably increase. Genome sequences of other great apes may prove particularly important, since changes in humans, which are absent in other hominids, are likely to be central to "what makes us human".

### Beyond a catalog of genomic features: searching for adaptive evolution

Comparative genomics is allowing us to assemble a catalog of genomic features unique to our species. Such a catalog, however, is not a description of the genetic basis of the human phenotype, as many of the genomic features identified may bear little functional relevance for understanding the human condition. How do we identify the subset of functionally important features? The most prominent approach has been to search for loci in the genome that show evidence of adaptive evolution [29]. Given that a history of adaptive evolution, by definition, indicates that a human-specific change in the genome has had functional implications, putatively adaptive loci are the strongest candidates for understanding the genetic basis of the human phenotype.

Several different signatures of adaptive evolution in the genome have been used to identify putatively adaptive loci. For protein-coding sequences, a powerful approach has been to compare the frequency of amino acid-changing, or nonsynonymous, substitutions at a locus (*K*_a_), to the frequency of synonymous substitutions (*K*_s_). Since most nonsynonymous substitutions are deleterious [30] whereas synonymous substitutions are generally neutral, the latter are much more likely to become common or fixed in a population, and *K*_a _is expected to be much smaller than *K*_s_. Nonsynonymous substitutions that confer an adaptive advantage, however, may rise very rapidly in frequency. Thus, a high *K*_a_/*K*_s _ratio at a locus is a potential signature of adaptive evolution [31]. This approach has been very successful in identifying the specific classes of genes in humans that are most strongly implicated in adaptive evolution. In a study of 7,645 orthologous genes between human and chimpanzee, analysis of *K*_a_/*K*_s _ratios found that genes subject to adaptive evolution in the human lineage are mainly involved in olfaction and nuclear transport [27]. More recently, analysis of 13,731 human-chimpanzee orthologs expanded this functional categorization to genes involved in sensory perception, immune response, apoptosis, and spermatogenesis [32]. Although genes expressed in the brain tend to be highly conserved, Dorus *et al. *showed that, nonetheless, this class of genes shows higher *K*_a_/*K*_s _ratios in humans than in other primate lineages, including the chimpanzee [33], and Yu *et al. *identified a subset of 47 genes, expressed in the brain, that show a particularly strong signature of adaptive evolution [28].

Although this approach can be very powerful, high rates of nonsynonymous substitutions may also result from a relaxation in selective constraint [34]. The stringent requirement of *K*_a_/*K*_s _> 1 overcomes this problem, as relaxed constraint will not elevate *K*_a _beyond *K*_s_. The detection power is then greatly reduced, however, since adaptive evolution at specific sites in a gene and along specific lineages, may be swamped by the effects of purifying selection across the gene as a whole and across most of the phylogeny. Consequently, several methods have been developed to estimate *K*_a_/*K*_s _ratios, either for individual portions of the gene or for individual lineages. For genes with well-defined functional domains, estimates can be made for each domain separately [35]. A gene may also be partitioned randomly into a set of windows, for which independent estimates are calculated [36]. Alternatively, site-specific methods have also been developed; these methods estimate the substitution rates of each individual site by using Maximum Likelihood (ML) models of a variable *K*_a_/*K*_s _ratio [37]. To estimate *K*_a_/*K*_s _ratios for individual lineages, one approach has been to infer the ancestral sequence by parsimony to allow pairwise comparison with each lineage separately [38]. Alternatively, branch-specific ML models have been developed, in which the *K*_a_/*K*_s _ratio is allowed to vary across individual branches in a phylogeny [39]. All of these methods increase our power to detect loci subject to adaptive evolution, either at a limited number of sites or across short evolutionary distances, while maintaining the stringency of *K*_a_*/K*_s _> 1. Recently, ML methods have also been developed for combining the site-specific and branch-specific models, to help detect episodes of adaptive evolution occurring at specific sites in a gene and along individual lineages [40].

A second approach for identifying signatures of adaptive evolution in coding sequences is to compare the ratios of synonymous to nonsynonymous substitutions between and within species. Under neutrality, these two ratios are expected to be equal, as interspecies divergence and intraspecies polymorphism are both linearly related to the neutral mutation rate. The McDonald-Kreitman tests look for an excess of nonsynonymous substitutions between species, relative to that found within a species. In a comparison of interspecies divergence to intraspecies polymorphism between human and macaque, Fay *et al. *estimated an accumulation of up to 1 adaptive substitution every 200 years since the divergence between humans and old world monkeys [41]. In a large-scale study of over 11,000 loci in humans and chimpanzees, Bustamante *et al. *identified 304 genes showing signatures of adaptive evolution [42]. Similar to Nielsen *et al.*'s Ka/Ks study, their findings also showed an overrepresentation of genes involved in immune response, gametogenesis, apoptosis, and sensory perception.

Other methods have been developed, based exclusively on intraspecies polymorphism, using reduced genetic diversity, high-frequency derived alleles, differentiation between populations, and high-frequency young haplotypes, as signatures of adaptive evolution in the very recent history of the species [43]. Though beyond the scope of this paper, it should be noted that polymorphism-based approaches are providing important complements to comparative genomic methods. Development of genome-wide datasets of human variation, such as the HapMap project [44] and the more restricted Seattle SNP database [45], will allow further implementation of population genetic approaches to the study of human evolution on a genomic scale.

For evolutionary analysis of gene duplications, far fewer analytical tools are available. Large interspecies differences in the sizes of gene families are often attributed to adaptive evolution [46]; this has been based mostly, however, on qualitative assumptions regarding the magnitude and frequency of the duplication events expected to persist in the population through neutral stochastic processes. The difficulty in assessing the significance of species differences in gene family size stems primarily from our lack of testable null models, making probabilistic statements impossible. Recently, Hahn *et al. *have demonstrated the utility of the stochastic birth-death process for modeling gene family evolution [47]. This model is based on continuous-time Markov processes, where states represent the current size of a population (gene copy number) and state transitions are defined by birth and death rates (the frequency of gene duplication and deletion events). Thus, specific hypotheses can be tested against a null model of random gene birth and death. Processes such as natural selection are predicted to violate the null birth-death model, causing extreme expansions or contractions in gene family size. This approach, though potentially very powerful, requires whole genome sequences of several closely related species, to test whether a gene family is evenly diffused across a phylogeny. Consequently, Hahn *et al. *used five closely related yeast species to demonstrate the utility of this approach, but its application to human evolution awaits the availability of additional primate whole genome sequences.

Over the past decade we've come to appreciate the potential functional importance of many noncoding sequences. Evolutionary analysis, however, is complicated by the difficulty in identifying functionally active noncoding elements. Because of the short divergence time between humans and chimpanzees, long-range conservation is not a good indicator of functional constraint. Several methods have recently shown great promise in moving the field forward. Heissig *et al. *compared the transcriptional activity of twelve promoters between humans and chimpanzees. Using a promoter assay in cell culture, seven of the twelve promoters were found to differ significantly between the two species, demonstrating the potential importance of promoter sequences in human evolution [48]. Searching for conservation between distantly related mammals and amniotes to identify potentially functional noncoding elements, Bush and Lahn found that many putative regulatory elements showed strong selective constraint between humans and chimpanzees [49]. Using a similar approach, Pollard *et al. *identified regions of the human genome showing lineage-specific acceleration. Of these, the most dramatic acceleration was found in a novel RNA gene that is expressed specifically in Cajal-Retzius neurons of the developing human neocortex, during cortical neuron specification and migration [50]. Donaldson and Göttgens used the consensus sequences of transcription factor binding sites to identify putative regulatory elements that are conserved between mouse and chimpanzee, but different in humans [51]. Their results showed that a significant proportion of human-chimpanzee sequence differences lie in these putative regulatory elements, suggesting that changes in transcriptional regulation has played an important role in shaping the human phenotype.

### From genotype to phenotype

The use of comparative genomics to answer what makes us human is inherently a reductionist approach. By breaking down the organism into a collection of nucleotide sequences, we can more easily ask what it is about our own sequence that makes us unique as a species. This approach is attractive, since evolution first and foremost acts at the level of DNA sequence. But the selective regime is imposed on the entire organism (or even more broadly on kin groups or populations), so the reductionist approach is a good starting point, but what we really want to understand is how it relates to constructing the human phenotype. Several approaches have been used to try and bridge the gap between genotype and phenotype, but as we'll discuss at the end, this challenge remains the biggest hurdle to overcome.

The use of human disease data in conjunction with comparative genomics has been a useful approach in taking the first step towards understanding the potential phenotypic consequences of an evolutionary change. The transcription factor *FOXP2 *has been implicated in the cognitive process underlying speech and language [52]. Evolutionary analysis of *FOXP2 *identified two human-specific amino acid changes, and showed that this gene has been subject to strong adaptive evolution in humans since the divergence between humans and chimpanzees [53]. This has led to the hypothesis that the evolution of *FOXP2 *may have contributed to the emergence of human language. *ASPM *and *Microcephalin *are two of six loci associated with autosomal recessive primary microcephaly, a developmental defect in which the overall architecture of the brain is preserved, but its volume is reduced three-fold to the size of the early hominid brain [54]. Both genes contain several human-specific amino acid changes, and a number of studies have identified strong signatures of adaptive evolution at these loci along the human lineage [55-58]. Given the atavistic phenotype of primary microcephaly, it has been suggested that these genes may have played a role in human encephalization.

An alternative approach has been to look at a gene's function based on model systems. Similar to *ASPM *and *Microcephalin*, the function of the neuropeptide PACAP in regulating cortical neural progenitor proliferation, and the identification of a highly accelerated evolutionary rate in humans, has suggested a role in human encephalization [59]. Unlike the primary microcephaly genes, however, the evolutionary study of PACAP was prompted by data from experiments in cell culture [60] and rat embryos [61], rather than human disease. For the myosin heavy chain gene *MYH16*, mutational analysis in the mouse [62], and its expression pattern in the masticatory muscles of the macaque monkey [63], prompted an investigation into the potential role of this gene in the evolution of human cranial muscle fibers. Comparative sequence analysis of *MYH16 *revealed a human-specific loss-of-function mutation, dating back about 2.4 million years. This has suggested a possible mechanism for the masticatory gracilization of the genus *Homo *during the Pleistocene [63].

Finally, in order to analyze the functional significance of evolutionary changes in regulatory elements, gene expression profiles have been studied extensively. Most studies to date have used microarray analysis, and the primary focus has been on gene expression in the brain [64-66]. These studies have generally arrived at two important conclusions: First, gene expression in the brain has been significantly upregulated during human evolution; and second, relatively few genes expressed in the brain show significant divergence between human and chimpanzee, compared to genes expressed in other tissues. A potential complication in these studies, however, is the spurious variation that is associated with analyses of tissue samples from a relatively small number of individuals. Variations in gene expression between individuals within a species, as well as between different cell types within a tissue sample, are potentially problematic. Consequently, Karaman *et al. *took an alternative approach, comparing patterns of gene expression between humans and African great apes in fibroblast cell lines, rather than tissue samples. Similar to other studies, their findings also revealed species-specific gene expression profiles. Some of the functional categories overrepresented included genes involved in the extracellular matrix, metabolic pathways, signal transduction, stress response, inherited overgrowth, and neurological disorders [67]. Alternatively, Popesco *et al. *used Western blotting and immunofluorescence to study gene expression profiles in human tissues, following a genome-wide survey of gene duplications, in which they found that the most striking human-specific amplification has been in DUF1220 protein domains [68]. These domains were shown to be highly expressed in regions of the brain associated with higher cognitive function, and to have a neuron-specific expression pattern in the brain.

### Future prospects

Prompted by the technological and theoretical advances of the molecular era, the use of comparative genomics in the study of human brain evolution marks a true paradigm shift. This reductionist approach is allowing us to reach unparalleled resolution, reframing our question in reference to the very unit that evolution itself operates on. A major limitation of the traditional approach, centered primarily on comparative neural anatomy, has been that important evolutionary changes are often difficult to detect on the phenotypic level, given the immense complexity of a systems-wide phenotype. Thus, the subtle, fine-scale anatomical differences between the human and chimpanzee brains, which undoubtedly belie our significant behavioral differences, are almost entirely obscured by the overwhelming similarity between them. Comparative genomics circumvents this problem, tackling the evolutionary novelty itself before it joins the massive network of interdependent phenotypes, each no longer separable from the rest.

But this approach is not without a price. Comparative genomics may provide the necessary resolution for identifying DNA sequence differences between humans and other species, but like any reductionist approach, ultimately we'll need to figure out how all the individual pieces fit together. In other words, how do we connect our findings with concrete phenotypes relevant to the human condition? Current efforts have mostly borrowed from the wealth of information coming out of genetic studies of human disease, mutational analysis of model organisms, and gene expression profiles. These approaches, though an essential starting point, can never be fully informative. Relying on indirect inference, rather than experimental validation, they still don't address the functional consequences of the specific evolutionary changes.

A true "bottoms-up" approach will require that we take our findings from comparative genomics back to the laboratory. *In vitro *assays, and ultimately the introduction of human transgenes into suitable model organisms, will be essential for us to begin connecting evolutionary genotypic modifications to their resultant phenotypic consequences. This necessity poses a major challenge for a field like comparative genomics, which inherently defines itself by its high-throughput capabilities. Comparative genomics is in its infancy, and already we face an increasingly narrower bottleneck, with continuous influx of data from one end and only single-gene approaches on the other. The next major challenge, therefore, will be to develop high-throughout methods for functional analysis of comparative genomic data. Only then will we be able to begin assembling a picture of how the varied multitude of human-specific features all fit together in a unified system. This is clearly a daunting task. But if, like Karl Pribram, we dare ask the age-old question that has preoccupied human imagination for millennia, then it will come as no surprise that the answer is not easy to come by.
